# Screening for adulticidal activity against Anopheles arabiensis in ten plants used as mosquito repellent in South Africa

**DOI:** 10.1186/1475-2875-13-173

**Published:** 2014-05-06

**Authors:** Edison J Mavundza, Rajendra Maharaj, Jude C Chukwujekwu, Jeffrey F Finnie, Johannes Van Staden

**Affiliations:** 1 Malaria Research Unit, Medical Research Council, 491 Peter Mokaba Ridge, Overport, Durban 4001, South Africa; 2 Research Centre for Plant Growth and Development, School of Life Sciences, University of KwaZulu-Natal Pietermaritzburg, Private Bag X01, Scottsville 3209, South Africa

**Keywords:** Malaria, Mosquitoes, *Anopheles arabiensis*, Adulticidal, *Aloe ferox*

## Abstract

**Background:**

Due to the development of resistance to synthetic insecticides, adverse effects to human health, non-target organisms and the environment, there is an urgent need to develop new insecticides, which are effective, safe, biodegrable and target-specific. This study was undertaken to evaluate the adulticidal activity of 10 plants used traditionally as mosquito repellents in South Africa.

**Methods:**

The dried plant materials were extracted with dichloromethane (DCM) and ethanol (EtOH). The extracts were evaluated for adulticidal activity against *Anopheles arabiensis* mosquitoes, a potent malaria vector in South Africa. Adult mortality was observed after 24 hours of exposure.

**Results:**

All the extracts showed adulticidal activity. The highest activity was observed in both DCM and EtOH extracts of *Aloe ferox* leaves with 98 and 86% mosquito mortality, respectively. The DCM extract of *A. ferox* leaves was then subjected to a dose-dependent bioassay to determine the EC_50_ value. The extract exhibited an EC_50_ value of 4.92 mg/ml.

**Conclusion:**

The results of the present study showed that the DCM extract of *A. ferox* leaves may have the potential to be used as an insecticide against *An. arabiensis*. Further studies to isolate and identify active compounds are in progress.

## Background

Mosquito-borne diseases, such as malaria, Japanese encephalitis, filariasis, dengue and yellow fever remain a major source of illness and death worldwide, particularly in tropical and subtropical countries [1]. It is estimated that more than 700 million people are infected with mosquito-transmitted diseases annually [2]. Among these diseases, malaria, which is caused by parasites of the genus *Plasmodium* and transmitted by the bite of infected female mosquitoes of the genus *Anopheles,* continues to be a major public health problem in tropical and subtropical countries, despite decades of control efforts. In 2010, the World Health Organization (WHO) estimated that there were 216 million cases of malaria and 655,000 deaths worldwide. About 91% of these deaths occurred in sub-Saharan Africa, and were mostly in children under five years of age [3].

Despite significant efforts to control malaria in South Africa since 1930 [4], the disease remains a serious health problem [5]. An estimated 4.3 million people are at risk of contracting malaria [4]. In 2000, the highest number (61,934) of malaria cases were reported, the worst levels of malaria recorded since the epidemics of the 1930s [6]. In South Africa, malaria is currently confined to the low-altitude regions of Limpopo, Mpumalanga and KwaZulu-Natal provinces, in the north-eastern part of the country, along the border with Mozambique and Swaziland. Malaria transmission in South Africa is distinctly seasonal [7,8], with *Anopheles arabiensis* being the major vector [5].

Since there is currently no effective vaccine available for the prevention of malaria, vector control is the main strategy used to control this disease. IRS (indoor residual spraying), which is the application of insecticides on the walls and ceilings of residential structures in order to kill and/or repel the adult vector mosquitoes that land and rest on these surfaces, is one of the primary vector control methods for reducing and interrupting malaria transmission [9]. Presently, IRS primarily depends on applications of synthetic insecticides. There are currently 12 insecticides belonging to four chemical groups recommended by WHO for IRS, namely, organochlorides, organophosphates, carbamates and pyrethroids. Among these insecticides, DDT (1,1,1-trichloro-2,2-bis (4-chlorophenyl) ethane), an organochloride, is the one with the longest residual efficacy (6–12 months depending on dosage and substrate) [9,10]. DDT was introduced into malaria control programmes in the 1940s [11], and it has been effective in reducing malaria morbidity and mortality in South Africa [12]. It also contributed to the eradication of malaria in the United States, Japan, Korea, Taiwan, Spain, Italy, the Balkans, Greece and Northern Africa during the Global Malaria Eradication Programme (GMEP) of 1955–1969 [13,14]. Despite its effectiveness in reducing malaria, the use of DDT has resulted in many problems such as, adverse effects on the environment, human health, non-target organisms, and the development of insecticide resistance in mosquito populations [15]. There is, therefore, an urgent need to develop new insecticides, which are effective, safe, biodegradable and target-specific.

Plants may be an alternative source of mosquito-control agents because they constitute a rich source of bioactive chemicals [16,17]. Natural products are generally preferred because of their less harmful nature to non-target organisms and due to their innate biodegradability [16,18]. Humans have used plants to control insects since time immemorial [19], even before the discovery of synthetic organic insecticides [20]. Much effort has, therefore, been focused on plant extracts or phytochemicals as potential sources of mosquito insecticidal agents or as lead compounds. Today, over 2000 plant species are known to possess insecticidal activities [21-23]. In view of the recently increased interest in developing plant-derived insecticides, the present study was undertaken to assess the adulticidal potential against *Anopheles arabiensis* of 10 extracts from the selected plants that are reportedly used traditionally as mosquito repellents in South Africa [24].

## Methods

### Plant collection

Plant materials (Table 1) were collected from Ndumo Village, in uMkhanyakude district, KwaZulu-Natal Province, South Africa. Voucher specimens were prepared and deposited at the Bews Herbarium, University of KwaZulu-Natal, Pietermaritzburg Campus.

**Table 1:** **Plants screened for adulticidal activity against *Anopheles arabiensis***

**Family**	**Botanical name**	**Common name**	**Local name**	**Voucher number**	**Part used**
Xanthorrhoeaceae	*Aloe ferox* Mill.	Cape aloe	iNhlaba	EM08	Leaves
Anacardiaceae	*Sclerocarya birrea* (A.Rich.) Hochst.	Marula	Umango	EM10	Leaves
Balanitaceae	*Balanites maughamii* Sprague.	Torchwood	uGobendlovu	EM09	Bark
Euphorbiaceae	*Croton menyaarthii* Pax	Rough-leaved Croton	Hubeshani	EM05	Leaves
Meliaceae	*Melia azedarach* L.	Chinaberry	Umsilinga	EM01	Leaves
Meliaceae	*Trichilia emitica* Vahl	Natal Mahogany	Umkhuhlu	EM06	Leaves
Olacaceae	*Olax dissitiflora* Oliver	Bastard Sourplum	Mampuzane	EM04	Bark
Rutaceae	*Clausena anisata (Willd.) Hook.F.*	Perdepis	Umsanga	EM02	Leaves
Sapindaceae	*Atalaya alata (Sim) H.H.L. Forbes*	Lebombo krantz Ash	Umnondo	EM07	Leaves
Verbenaceae	*Lippia javanica* (Brum.f) Spreng.	Fever tea	Umsuzwane	EM03	Leaves

### Preparation of plant extracts

Plant materials were dried in an oven at 30–60°C. The drying time and temperature varied depending on the nature of the plant material. The dried plant materials were ground into powders by an electrical blender and stored in airtight containers under dark conditions at room temperature. The ground plant materials were extracted separately with 20 ml/g of ethanol (EtOH) and dichloromethane (DCM) by sonication for 1 hour. The extracts were filtered through Whatman No. 1 filter paper and concentrated under vacuum using a rotary evaporator (Büchi, Germany) at 30°C. The concentrated extracts were dried at room temperature under a stream of cold air. The dried extracts were stored at 4°C in the dark until required for assays.

### Rearing mosquitoes

The adulticidal activity of plant extracts was evaluated using laboratory-reared *An. arabiensis* mosquitoes, a potent malaria vector in South Africa. The mosquitoes were obtained from a permanent colony maintained at 27 ± 2°C and 85% relative humidity in the insectary of the Malaria Research Unit, Medical Research Council, Durban, South Africa. Larvae were fed on dog biscuits and yeast powder at a 3:1 ratio. Adults were provided with a 10% sucrose solution. Female mosquitoes were periodically blood-fed on restrained albino guinea pigs for egg production. The guinea pigs were reared according to the National Research Council's guidelines for the care and use of laboratory animals [25].

### Adulticidal assay

The adulticidal activity of the plant extracts was evaluated following the WHO standard method with slight modifications [26]. Briefly, plant extracts were dissolved in acetone to prepare a testing concentration of 10 mg/ml. Two and half millilitres (2.5 ml) of testing concentration was impregnated into Whatman No 1. filter papers (12 × 15 cm). Acetone was used as a negative control while deltametrin (K-Othrine) was used as a positive control. The impregnated papers were air dried for 5 minutes and then inserted into an exposure tube in the WHO testing kit. Twenty, 2–5 day old, blood-starved female mosquitoes were introduced into the holding tube and held for 1 hour to acclimatize. The mosquitoes were then transferred by gentle blowing in the exposure tube. After 1 hour in the exposure tube, mosquitoes were then transferred back to the holding tube to recover. A pad of cotton soaked with 10% glucose solution was placed on the mesh screen to feed recovering mosquitoes. At the end of the 24 hour recovery period, the number of dead mosquitoes was recorded and the percentage mortality was calculated. Each extract was tested in duplicate and the assay was repeated three times.

## Results and discussion

**Figure 1:**
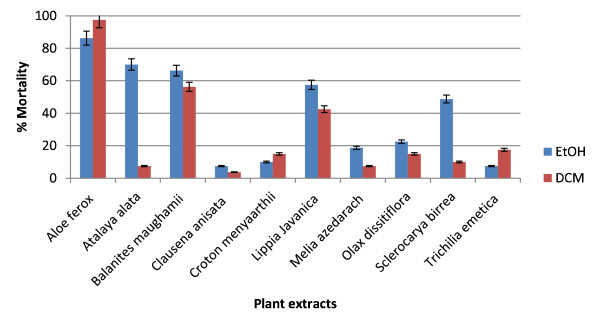
**Adulticidal activity of DCM and EtOH extracts against *An. arabiensis *** **at 10 mg/ml.**

The results of the adulticidal activities against *An. arabiensis* of dichloromethane and ethanol extracts of 10 plants that are used as mosquito repellents in South Africa are presented in Figure 1. All the extracts showed adulticidal activity after 24 hours of exposure with mosquito mortality ranging from 4 to 98%. Three levels were used to define the activity of extracts: 1- 49% low, 50-69% moderate and 70-100% high. Of the highly active extracts, the DCM extract of *Aloe ferox* leaves exhibited the highest activity with 98% adult mortality, followed by EtOH extracts of *A. ferox* leaves (86%) and *Atalaya alata* (70%). No activity was observed in the negative control, while the positive control exhibited 100% adult mortality. The high adulticidal activity shown by DCM extract of *A. ferox* leaves against *An. arabiensis* is not surprising since it has been reported as a multipurpose traditional medicine. The plant is traditionally used as a laxative, emetics, to treat arthritis, sinusitis, conjunctivitis, opthalmia, herpes, shingles, sore throat, red water hypertensions, infertility in women and impotance in men. Furthermore, it has also been reported to possess antioxidant, antimicrobial, anti-inflammatory, anticancer, antimalarial, and anthelmintic activities [27].

These findings are comparable to those of Nathan *et al*. [28], who reported the adulticidal activity of methanol extract of *Dysoxylum malabaricum* leaves against *Anopheles stephensi*. The adulticidal activity of ethanol extract of *Apium graveolens* seeds against *Aedes aegypti* has been reported [29]. Kovendan *et al*. [30] found the adulticidal activity of methanol extract of *Acalypha alinifolia* leaves against three mosquito species, *Ae. aegypti*, *An. stephensi* and *Culex quinquefasciatus.* Other plant species that are reported to possess adulticidal activity includes: *Curcuma aromatic* against *Ae. aegypti*[31]; *Aristolocia indica*, *Dolichos biflorus* and *Zingiber zerumbet* against *Culex gelidus* and Cx. *quinquefasciatus*[32]; and *Melia azedarach* against *An. stephensi*[33].

**Figure 2:**
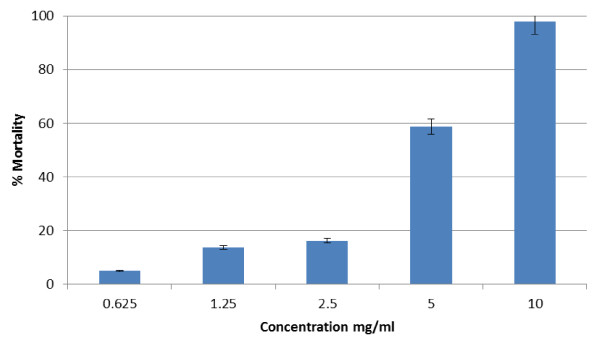
**Dose–response assay of DCM extract of *A. ferox *** **against *An. arabiensis.***

Due to its high activity, the DCM extract of *A. ferox* leaves was then subjected to a dose-dependent bioassay to determine the EC_50_ value. The extract was tested at five concentrations ranging from 0.6 to 10 mg/ml. After 24 hours of exposure, mosquito mortality ranging from 5 to 100% was observed (Figure 2) and an EC_50_ value of 4.92 mg/ml was recorded. The activity of this extract may be due to various compounds, such as phenolics, terpenoids, and alkaloids that exist in plants, and they may jointly or individually contribute to the insecticidal, ovicidal, repellent and antifeeding activities against various insect species [34]. *Aloe ferox* has been reported to contain compounds such as chromones, anthraquinones, anthrones, anthone-C-glycosides [27]. Therefore, the observed adulticidal activity of the DCM extract of *A. ferox* leaves may be attributed to these compounds. The adulticidal activity of the DCM extract of *A. ferox* against *An. arabiensis* is reported for the first time in this study.

## Conclusions

The obtained results indicate that the DCM extract of *A. ferox* leaves has potential to be developed as an insecticide against *An. arabiensis* mosquitoes. However, further studies to evaluate its toxicity and effects on non-target organisms and the environment need to be conducted. Studies aimed at isolation and identification of active compounds are in progress. Evaluation of adulticidal activity of the DCM extract of *A. ferox* leaves against other medical-important mosquito species is also considered. The results of the present study could be useful in promoting research aimed at the development of new agents for mosquito control based on bioactive chemical compounds from indigenous plant sources.

## Competing interests

The authors declare that they have no competing interests.

## Authors’ contributions

EJM conducted the experiments and wrote the manuscript. RM, JCC, JFF and JVS provided scientific inputs. All authors read and approved the manuscript.
